# Optimization of In Vitro *Mycobacterium avium* and *Mycobacterium* *intracellulare* Growth Assays for Therapeutic Development

**DOI:** 10.3390/microorganisms7020042

**Published:** 2019-02-01

**Authors:** Lauren Auster, Morgan Sutton, Mary Chandler Gwin, Christopher Nitkin, Tracey L. Bonfield

**Affiliations:** 1 Division of Pulmonology, Allergy and Immunology, Department of Pediatrics, Case Western Reserve University School of Medicine, 10900 Euclid Ave. BRB 822, Cleveland, OH 44106, USA; Lauren.Auster@case.edu (L.A.); mxs1175@case.edu (M.S.); mary.gwin@yale.edu (M.C.G.); crnitkin@chm.edu (C.N.); 2 Division of Neonatology, Department of Pediatrics, Children’s Mercy Hospital, University of Missouri-Kansas City School of Medicine, 2401 Gillham Rd., Kansas City, MO 64108, USA

**Keywords:** *Mycobacterium intracellulare*, *Mycobacterium avium*, optimized growth, anti-non-tuberculous mycobacterium therapeutic testing

## Abstract

Infection with nontuberculous mycobacteria (NTM) is a complication of lung disease in immunocompromised patients, including those with human immunodeficiency virus and acquired immune deficiency syndrome (HIV/AIDS), chronic obstructive pulmonary disease (COPD), and cystic fibrosis (CF). The most widespread, disease-causing NTM is *Mycobacterium avium* complex (*MAC*), which colonizes the lungs as a combination of *Mycobacterium avium*, *Mycobacterium intracellulare*, and other mycobacterial species. While combination drug therapy exists for *MAC* colonization, there is no cure. Therapeutic development to treat *MAC* has been difficult because of the slow-growing nature of the bacterial complex, limiting the ability to characterize the bacteria’s growth in response to new therapeutics. The development of a technology that allows observation of both the *MAC* predominant strains and *MAC* could provide a means to develop new therapeutics to treat NTM. We have developed a new methodology in which *M. avium* and *M. intracellulare* can be optimally grown in short term culture to study each strain independently and in combination, as a monitor of growth kinetics and efficient therapeutic testing protocols.

## 1. Introduction

Mycobacterial species not belonging to *Mycobacterium tuberculosis* or *Mycobacterium leprae* are designated nontuberculous mycobacteria (NTM). NTM are pervasive in the environment and contribute to pulmonary disease in immunocompromised patients including diseases such as cystic fibrosis (CF) and acquired immunodeficiency syndrome (AIDS) [1]. The most common, disease-causing NTM is *Mycobacterium avium* complex (*MAC*), a slow-growing symbiotic combination of *Mycobacterium avium* and *Mycobacterium intracellulare*, with other non-tuberculosis species being least dominant [1,2]. Due to the emergence of the AIDS epidemic, contracting HIV is the most significant risk factor for *MAC* infection [3]. Infection with NTM is also common in patients with CF, including *M. avium* and *M. intracellulare,* the latter being more prominent in severe pulmonary disease [4]. Further, patients with a history of lung disease, such as chronic obstructive pulmonary disease (COPD), tuberculosis (TB), or pneumonia [5,6] and idiopathic pulmonary fibrosis (IPF) demonstrated an increased propensity for NTM colonization [7].

Traditionally, NTM and *MAC* therapeutic testing studies have focused on *M. avium*, not particularly separating *M. avium* from *M. intracellulare* [8,9]. Drug therapy for *MAC* infection includes gentamicin, clarithromycin, azithromycin, rifampin, rifabutin, ethambutol, and streptomycin [10]. Unfortunately, treatment of *MAC* often yields poor results, possibly due to the toxicity of higher dosages of these drugs and their inherent pharmacology [11]. The limitations of dosage based on toxicity can result in poor therapeutic testing sensitivity as well as an inability to monitor multiple therapeutic treatment drug interactions. Patients with *M. intracellulare* lung disease tend to present with more severe infection symptoms and *M. intracellulare* prognosis than those with *M. avium* lung disease, suggesting that therapeutic development which might differentiate or distinguish the two strains would be therapeutically beneficial [12], with patient-specific therapeutic application done post-colonization identification. Since methods that compare *M. avium* and *M. intracellulare* are currently time consuming and not necessarily pathogen specific [13], we developed a new and innovative protocol which can speed up the growth kinetics for therapeutic testing in short term in vitro quantitative assays. *M. intracellulare, M. avium*, and *MAC* [8,9,14] typically take 2–3 weeks to grow in the traditional solid media phase, whereas broth media can produce more rapid results [1]. Both methods use media optimized for mycobacterial growth, most commonly 7H9 broth and 7H10 agar plates [1,8,9,14]. In these studies, we utilized gentamicin as a sub-optimal antibiotic to monitor the sensitivity of the assays overall. Since *MAC* is primarily composed of *M. avium* and *M. intracellulare* [1,2], our protocol demonstrates the capability to optimize the growth and characterization of each of these strains independently and in combination for systematic development of therapeutic drug testing using cost-effective, highly sensitive quantitative measures of growth assessment.

## 2. Materials and Methods

### 2.1. Preparation of 7H9 Broth with ADC Enrichment

Broth was prepared according to American Type Culture Collection (ATCC) guidelines [15]. To make the initial 7H9 broth, 2.35 g 7H9 broth base, 2.0 mL glycerol, and 450 mL deionized water were combined in a glass bottle and autoclaved on a liquid cycle. After cooling to 45–50 °C, 50 mL of ADC Enrichment was aseptically added and mixed. The entire 7H9 broth with ADC Enrichment was filter sterilized and stored in a large, glass round bottle at 2–8 °C prior to utilization in monitoring colony forming units (CFUs) of each pathogen. All culture conditions were done at homeostatic pH 7.5 to minimize the variability of pathogen response.

### 2.2. Initial Bacterial Preparation and Preservation

Freeze-dried *M. avium* and *M. intracellulare* were removed from packaging using the double-vial preparation instructional guide from the ATCC [16]. Each pellet was rehydrated by adding 0.5–1 mL of enriched 7H9 broth from an individual 5–6 mL aliquot of broth and then aseptically transferred into the aliquot flask after mixing. The bacterial flasks were incubated at 37 °C in a shaking incubator for one week, for stabilization in culture prior to initiation into the therapeutic testing protocols or freezing protocols for future studies. All studies were standardized against growth medium as the baseline control.

### 2.3. Optical Density

Optical density (OD) was measured using the Beckman DU 530 UV/VIS Spectrophotometer (Beckman Coulter, Brea, CA) at each time point throughout the experiment. Five time points at 18 h apart were chosen based on generation of growth curves from start to cessation of growth. Samples (1 mL) from each bacterial flask were transferred to cuvettes for the ability to refract light at 600 nm and returned to their respective flasks. OD provides insight into the bacterial growth prior to receiving CFU data (see Section 2.4 Colony Forming Units). Comparisons were made between the variables using the culture medium as a control.

### 2.4. Colony Forming Units

Colony forming units (CFU) were measured one time point per day throughout each of the individual experiments. The bacteria were evaluated over several dilutions to define the best end-point for quantitative measurement. The final studies utilized 10^−6^ and 10^−7^ dilution in phosphate buffered saline (PBS) and were grown in duplicate (10 µL/column) on 7H10 Middlebrook agar plates (Teknova, Hollister, CA, USA). The plates were incubated at 37 °C for one week followed by an assessment of CFUs by at least two different counters. Comparisons were made utilizing the culture medium as a control.

### 2.5. Resazurin Assay

The kinetics of bacterial growth was monitored using the CellTiter-Blue ® Cell Viability Assay [17]. Bacterial samples collected from each time point and monitored in triplicate (50 µL per well) using a 96 well with background controls of PBS and 7H9 broth. After diluting 1:1 with PBS, 20 µL cell titer blue was added to quantify bacterial growth by fluorescence using corresponding SoftMax Pro 6.5.1 software (Molecular Devices, Sunnyvale, CA, USA). Comparisons were made utilizing the culture medium as a control.

### 2.6. Gentamicin Studies

Gentamicin was chosen for therapeutic testing in the resazurin, OD, and CFU assays to establish the lower end specificity and sensitivity between presence and absence of a therapeutic in the newly developed growth protocol. *M. intracellulare*, *M. avium*, and *MAC* were grown in the presence and absence of 0.1, 0.5, 1, 5, 10, 20 µg/mL gentamicin, with antibiotics added on the first day of the 90-hour culture. All comparisons were done against bacteria grown in the basal medium without antibiotics or the culture medium without pathogens.

### 2.7. Statistics

Two-tailed, paired *T*-test analysis was performed to determine the statistical significance of each assay. All studies were standardized against the growth medium as the baseline control. For the gentamicin dose-response study, two-tailed, paired *T*-test and linear regression analyses were used to identify significance of both the rate of growth and total growth over 90 h, respectively. For therapeutic testing, ANOVA and linear regression analysis were utilized to correlate the different assay formats and end-points. Graphics and statistics were done using GraphPad Prism 7 software (GraphPad Software, San Diego, CA, USA). Asterisks above bars denote statistical significance in each of the datasets.

## 3. Results

### 3.1. Optical Density Indicates Increased Bacterial Concentration over 90 h

**Figure 1:**
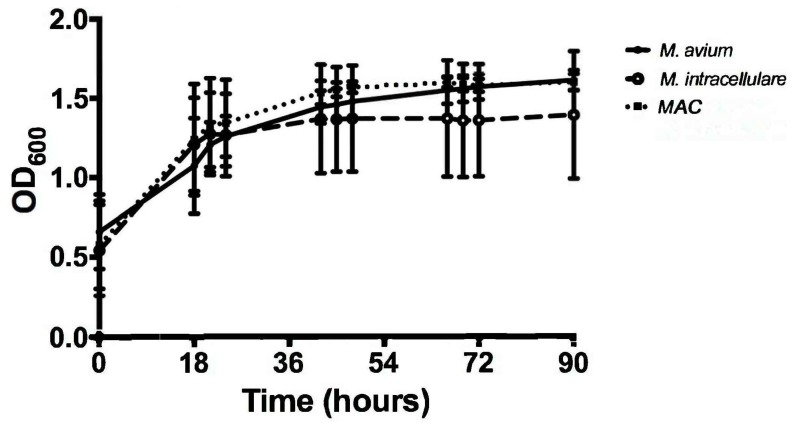
Optical density (OD) kinetics of *M. avium* (solid line, solid symbol), *M. intracellulare* (dashed line, open symbol), and *MAC* (*M. avium* combined with *M. intracellulare*) (dotted line with solid square symbol) over 90 h at 600 nm, *n* = 7.

**Figure 2:**
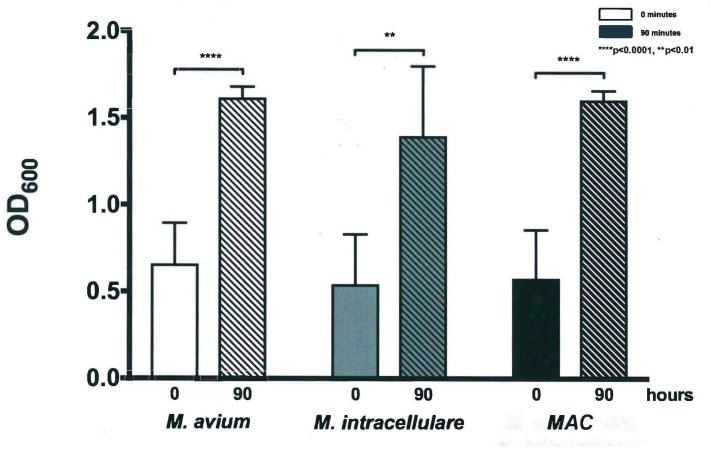
Optical density of *M. avium*, *M. intracellulare*, and *MAC* (*M. avium* combined with *M. intracellulare*) at time zero and 90 h. *M. avium* and *MAC* (*M. avium* with *M. intracellulare*) demonstrated increased OD at a significance of **** *p* < 0.0001 according to two-tailed, paired *T*-test analysis. The increased OD of *M. intracellulare* was also significant, but at ** *p* < 0.01, *n* = 7.

The optical density (OD) of samples from each bacterial strain and combination were obtained at several time points spread over a 90-hour incubation period. The 90-hour incubation period was chosen due to monitoring of growth kinetics to the cessation point, with 18-hour increments chosen to demonstrate appreciable changes. Using these OD measurements, the growth of M. avium, M. intracellulare, and MAC were evaluated. The two different bacteria demonstrated a similar kinetic trend and increased over 90 h, as shown in Figure 1. Variation between the bacterial strains was minimal, especially up to 45 h, but each strain exhibited a significant increase in OD after 90 h when compared to the baseline value at time zero (P < 0.1) as shown in Figure 2. M. avium and MAC had less variability in OD and reached higher OD values than M. intracellulare by the conclusion of the experiment, as shown in Figure 1. These studies demonstrate that M. avium and MAC demonstrate more stable growth kinetics than M. intracellulare alone, as defined by less variability and higher OD levels. Over the course of the studies, the OD levels reached saturation, related to the combination of live versus dead bacteria and the ability to refract light. With the limitations and lack of specificity of OD, each time point was also followed closely with CFUs (see Section 3.2) throughout the duration of the experiment. This provided the opportunity to define differences between the limitation of detection method and growth cessation.

### 3.2. Colony Forming Units Confirms Bacterial Growth over 90 h

**Figure 3:**
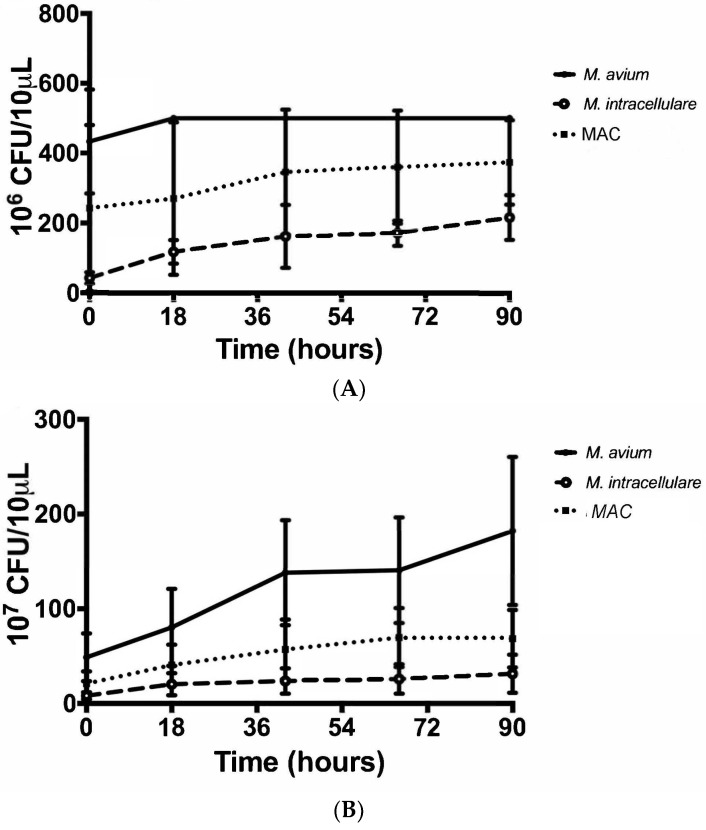

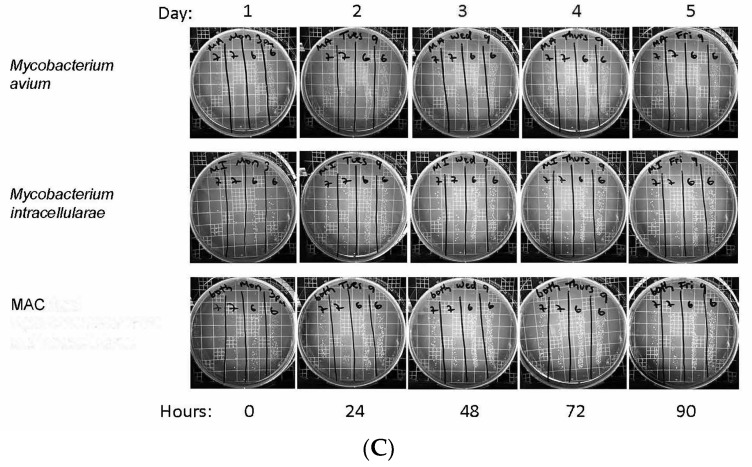
Bacterial growth kinetics of *M. avium*, *M. intracellulare*, and *MAC* (*M. avium* combined with *M. intracellulare*) over 90 h at (**A**) 10^−6^ dilutions (*n* = 7); and (**B**) 10^−7^ dilutions (*n* = 7). (**C**) Agarose plates of *M. avium*, *M. intracellulare*, and *M. avium* combined with *M. intracellulare* at 10^−6^ and 10^−7^ dilutions.

Colony forming units (CFU) were measured daily throughout the experiment. Dilutions were plated out to both 10^−6^ and 10^−7^, which enabled bacterial colonies to be counted at different concentrations to determine density impact of the new culturing technology on growth and bacteria phenotypes, as shown in Figure 3. At 10^−5^ dilutions, colonies were too numerous to count (TNTC) while at 10^−8^ dilutions, no growth was observed. Several dilutions were done during each experiment to gauge the overall impact of the bacterial concentration on CFUs. CFUs that reach a value that is too numerous to count (TNTC) within the manuscript are noted as 500 CFU. At 10^−6^ dilutions, *M. avium* exhibited high CFUs and leveled off at 500 CFU after only 20 h of growth, as shown in Figure 3A. *M. intracellulare* and *MAC* demonstrated a steady increase in CFUs over 90 h, but with some variability, as shown in Figure 3A. Similar to the differences seen in OD, *M. avium* conferred higher CFUs compared to *M. intracellulare*, most likely due to its dominant role in *MAC*. At 10^−7^ dilutions, the kinetics of *M. avium* were more easily observed and indicated a steady increase in CFUs over 90 h, as shown in Figure 3B. *M. intracellulare* and *MAC* also had increased CFUs, but at a slower rate over the defined dilutions. *M. avium* has more robust growth kinetics than *M. intracellulare*, so it is plausible that the slower rate of *MAC* growth over the defined dilutions is due to the combination of the faster and slower growing strains. Measuring CFUs at both 10^−6^ and 10^−7^ dilutions was pertinent to fully characterizing the growth and the phenotype of the bacteria, as shown in Figure 3C, and to make comparisons between the different bacterial strains and combinations if the colonies ultimately change or look different.

**Figure 4:**
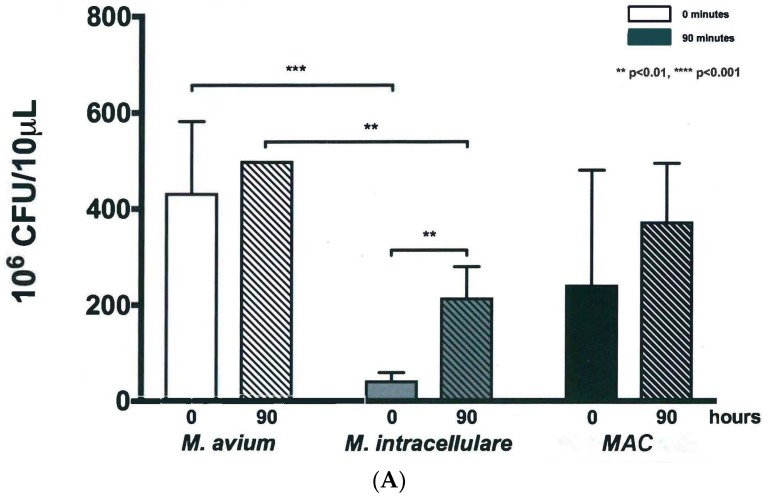

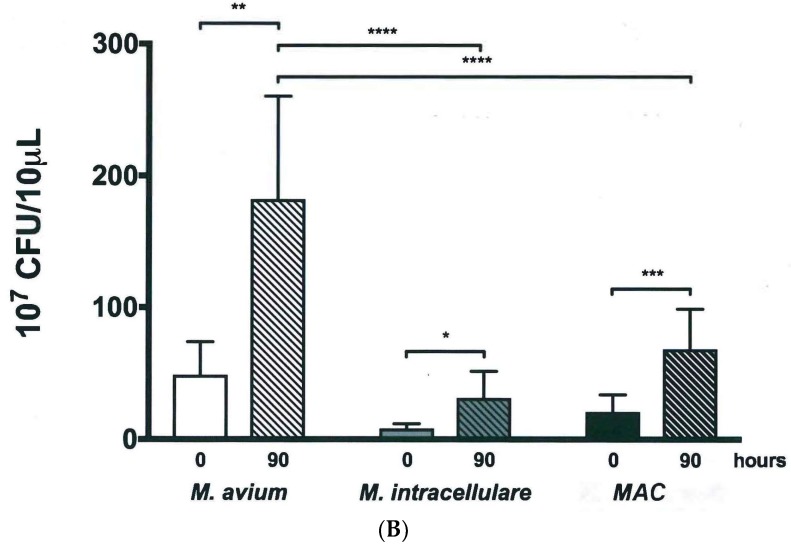
Bacterial growth of *M. avium*, *M. intracellulare*, and *MAC* (*M. avium* combined with *M. intracellulare*) at time zero and 90 h at (**A**) 10^−6^ dilutions. Increased colony forming units (CFUs) after 90 h were significant for *M. intracellulare* at ** *p* < 0.01 according to two-tailed, paired *T*-test analysis. Growth of *M. avium* and *MAC* (*M. avium* with *M. intracellulare*) after 90 h was not significant at this dilution. The CFU differences between *M. avium* and *M. intracellulare* were significant at time zero with *** *p* < 0.001 and at 90 h with ** *p* < 0.01. (**B**) 10^−7^ dilutions. Increased CFUs after 90 h were significant for *M. avium* at ** *p* < 0.01, *M. intracellulare* at * *p* < 0.05, and *MAC* (*M. avium* with *M. intracellulare*) at *** *p* < 0.001 according to two-tailed, paired *T*-test analysis. The CFU differences between *M. avium* and *M. intracellulare*, and between *M. avium* and *MAC* (*M. avium* with *M. intracellulare*), were significant at 90 h with **** *p* < 0.0001, *n* = 7.

The differences observed between dilutions when comparing CFUs at time zero and after 90 h for all three groups demonstrated several differences, specifically when you compare the growth at the start of the assay (0 minutes, solid bars) to the final growth at the end of the study (90 minutes, hatched bars). An increase in CFU of *M. intracellulare* was detected at a (** *p* < 0.01) at 10^−6^ and 10^−7^ dilutions (*p* < 0.05) while CFU increases were not significant for *M. avium* and *MAC* 10^-6^, as shown in Figure 4A. At 10^−7^ dilutions, the growth of *M. avium* and *MAC* after 90 h was significant (*p* < 0.1), while growth of *M. intracellulare* alone was significant (*p* < 0.05), as shown in Figure 4B. We noted that the variability in CFUs, particularly for *MAC,* could be influenced by the recycling of bacteria from week to week after they were transferred to new flasks of broth for subsequent experiments. Furthermore, when the bacteria appeared to be growing less effectively after multiple passages, fresh flasks of bacteria were started using frozen aliquots. Overall, *M. avium*, *M. intracellulare*, and *MAC* exhibited increased CFUs over a 90-hour period, indicating growth and colony formation.

### 3.3. Resazurin Assay Detects Metabolic Activity of the NTM

**Figure 5:**
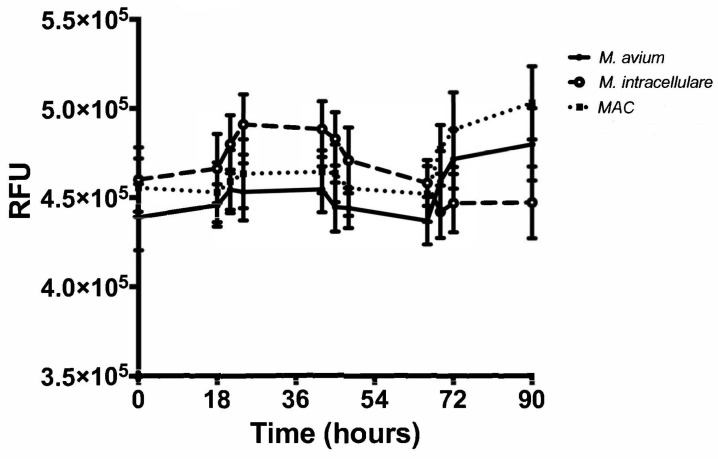
Cellular metabolic activity of *M. avium*, *M. intracellulare*, and *MAC* (*M. avium* combined with *M. intracellulare*) over 90 h. A cyclic trend was observed, *n* = 7.

**Figure 6:**
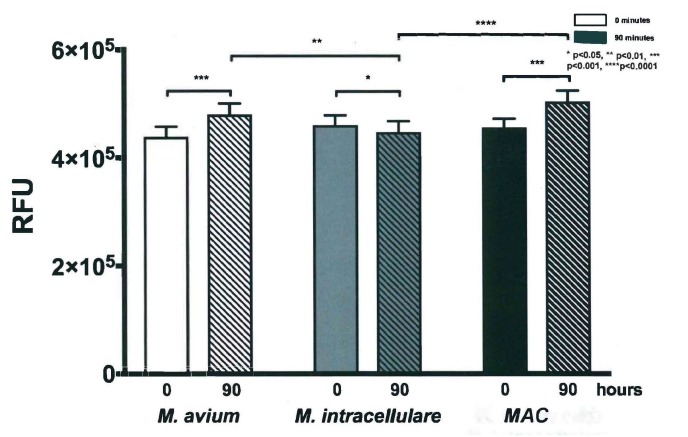
Cellular metabolic activity of *M. avium*, *M. intracellulare*, and *MAC* (*M. avium* combined with *M. intracellulare*) at time zero and 90 h. Increased activity after 90 h was significant for *M. avium* at *** *p* < 0.001, *M. intracellulare* at * *p* < 0.05, and *MAC* (*M. avium* with *M. intracellulare*) at *** *p* < 0.001 according to two-tailed, paired *T*-test analysis. Significant differences after 90 h were observed between *M. avium* and *M. intracellulare* at ** *p* < 0.01 and between *M. intracellulare* and *MAC* (*M. avium* with *M. intracellulare*) at **** *p* < 0.0001, *n* = 7.

The resazurin assay was performed at the end of each experiment to determine the metabolic activity of each bacterial strain at each time point along the course of the experiment. Samples from each time point throughout the experiment were saved and stored at 4 °C to be used in the assay. Higher levels of fluorescence indicated greater metabolic activity, relative to the stress of the availability of growth medium and potentially the introduction of growth inhibitors [18]. For all three strains and combinations, metabolic activity appeared to be cyclically consistent with changes in bacterial metabolism associated with cell cycle, as shown in Figure 5. This fluctuation of growth indicates intercellular activity as the bacteria propagated in culture over time [19]. Although metabolic activity fluctuated, fluorescence increased for both *M. avium* and *MAC* (*p* < 0.01), while it decreased for *M. intracellulare* (*p* < 0.05). However, when a direct comparison was made between the start of the assay and the end of the assay between *M. avium*, *M. intracellulare,* and the combined *MAC,* the *M. intracellulare* had a modest change, with increases in the *M. avium*-containing cultures (Figure 6). The difference between the bacteria combinations in overall growth may be related to the dominance and growth stability of *M. avium* compared to *M. intracellulare* or the growth condition optimization protocol. *M. intracellulare* may require modifications in growth conditions, which is an on-going focus in our laboratory.

### 3.4. Validation of New Assays to Monitor Therapeutics

**Figure 7:**
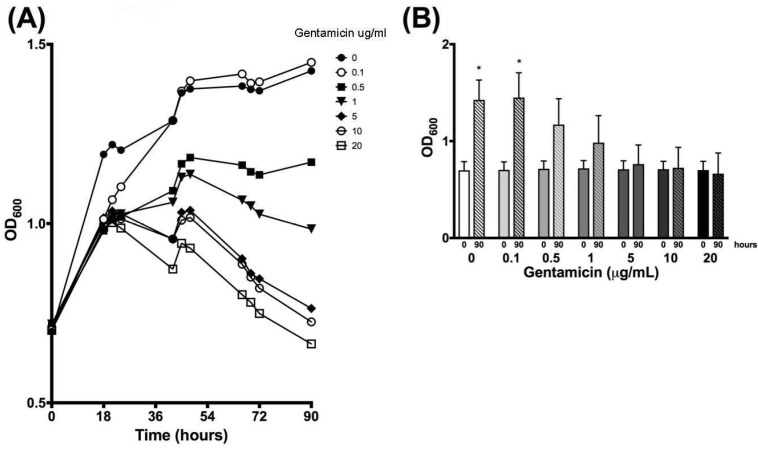
Optical density (OD) assay of *M. avium* gentamicin dose response. (**A**) OD of *M. avium* with gentamicin treatment over 90 h at 600 nm. Differences between the slopes of each treatment are significant at *p* < 0.0001 according to linear regression analysis. Average values are reported without error bars for graph clarity. (**B**) Optical density (OD) of *M. avium* with gentamicin treatment at time zero and 90 h. *M. avium* demonstrated increased OD with 0 and 0.1 µg/mL gentamicin treatment at * *p* < 0.05 according to two-tailed, paired *T*-test analysis. *M. avium* treated with higher doses of gentamicin were not significant, *n* = 3.

**Figure 8:**
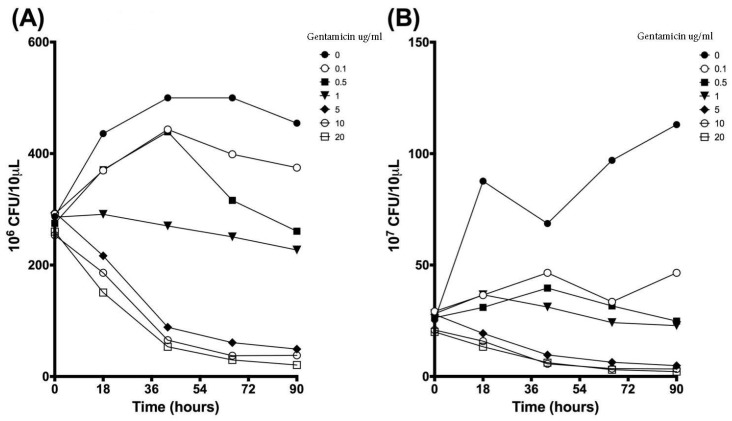
Growth kinetics of *M. avium* with gentamicin treatment over 90 h at (**A**) 10^−6^ dilutions. Differences between the slopes of each treatment are significant at *p* < 0.01 according to linear regression analysis. Average values are reported without error bars for graph clarity. (**B**) 10^−7^ dilutions. Differences between the slopes of each treatment are significant at *p* < 0.0001 according to linear regression analysis, *n* = 3.

**Figure 9:**
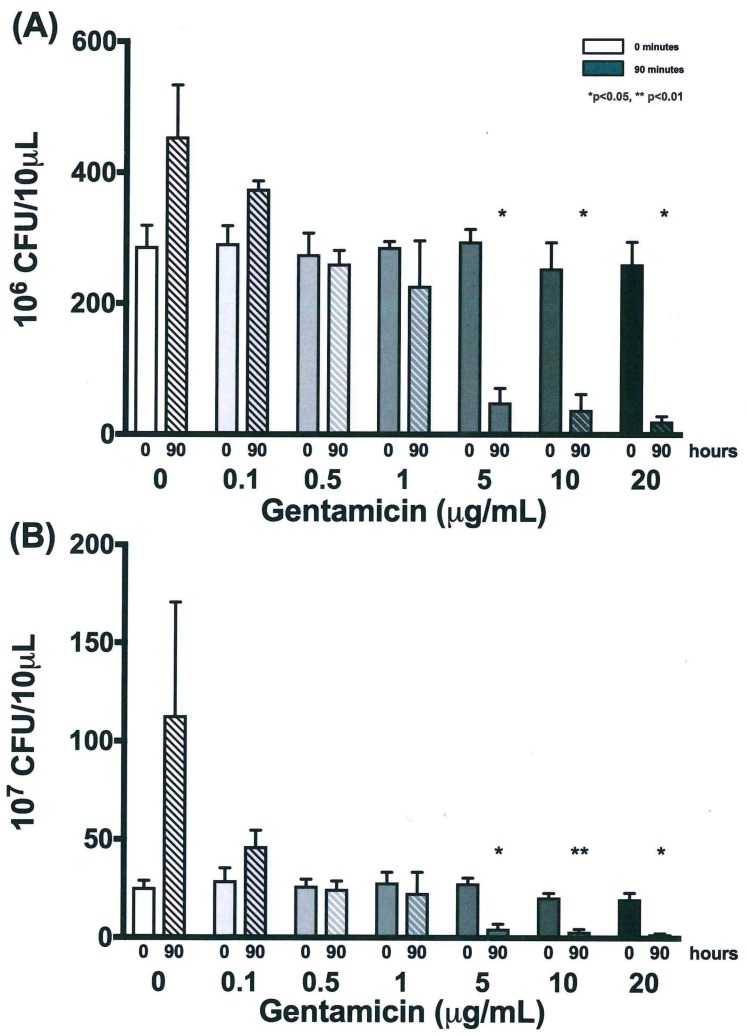
Growth of *M. avium* with gentamicin treatment at time zero and 90 h using colony forming units at (**A**) 10^−6^ dilutions. Decreased CFUs after 90 h for 5, 10, and 20 µg/mL gentamicin treatments were significant at * *p* < 0.05 according to two-tailed, paired *T*-test analysis. *M. avium* treated with lower doses of gentamicin were not significant. (**B**) 10^−7^ dilutions. Decreased CFUs after 90 h were significant for 5 and 20 µg/mL gentamicin treatments at * *p* < 0.05 and for 10 µg/mL gentamicin treatment at ** *p* < 0.01 according to two-tailed, paired *T*-test analysis. *M. avium* treated with lower doses of gentamicin were not significant, *n* = 3.

**Figure 10:**
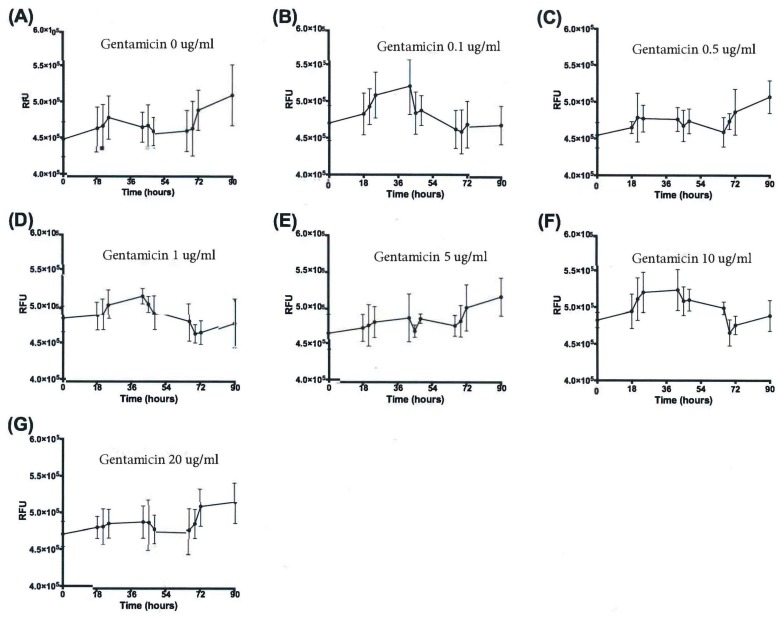
Cellular metabolic activity of *M. avium* with gentamicin treatment over 90 h. (**A**) 0 µg/mL gentamicin. (**B**) 0.1 µg/mL gentamicin. (**C**) 0.5 µg/mL gentamicin. (**D**) 1 µg/mL gentamicin. (**E**) 5 µg/mL gentamicin. (**F**) 10 µg/mL gentamicin. (**G**) 20 µg/mL gentamicin. Differences between the slopes of each treatment are significant at *p* < 0.001 according to linear regression analysis, *n* = 3.

**Figure 11:**
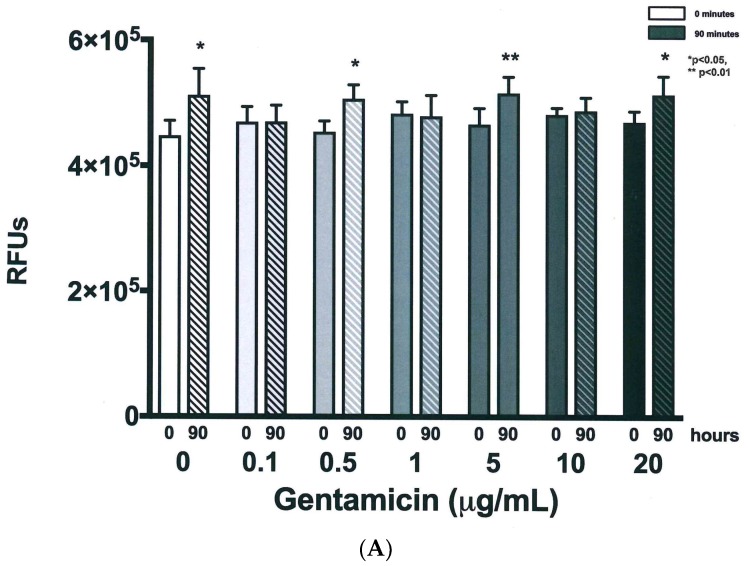

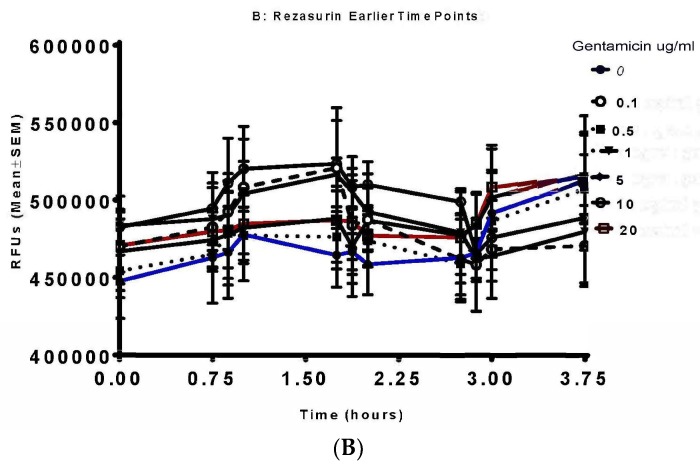
Cellular metabolic activity of *M. avium* with gentamicin treatment at time zero and 90 h. (**A**) Increased activity after 90 h was significant for 0, 0.5, and 20 µg/mL at * *p* < 0.05 and for 5 µg/mL at ** *p* < 0.01 according to two-tailed, paired *T*-test analysis when comparing to the initial 0-minute time point, *n* = 3. (**B**) The cyclic nature of the metabolism of *M. avium* over the course of the study in the presence and absence of the dose escalation of gentamicin. The blue line is without gentamicin, the red line is with the 20 µg/mL gentamicin dose.

The next focus of the work was to determine how the established growth conditions would impact the ability to detect the impact of anti-NTM therapeutics. *M. avium*, *M. intracellulare,* and the combined *MAC* were cultured in the presence and absence of a sub-optimal antibiotic (gentamicin). The purpose for picking gentamicin was to be able to establish sensitivity and specificity of the new NTM assays, as well as to enable the utilization of combined therapeutics for enhancing current antibiotic sensitivity. *M. avium* OD, CFUs, and the resazurin assay ultimately revealed distinct differences of detection when treated with dose-escalated concentrations of gentamicin. As the gentamicin dose was increased to 0.5 µg/mL and above, OD returned almost to baseline levels after 90 h. Differences in OD kinetics of each gentamicin treatment were significant according to linear regression analysis, as shown in Figure 7A. To maintain figure clarity, OD mean and standard deviation values are listed in Table 1. At the end of the experiment, significant increases in OD were observed for only the 0 and 0.1 µg/mL gentamicin treatments, while higher gentamicin doses stunted further *M. avium* growth, as shown in Figure 7B. CFU kinetics displayed a similar trend at both 10^−6^ and 10^−7^ dilutions, with increased gentamicin doses resulting in decreased or stunted bacterial growth. Differences in CFU growth kinetics were significant according to linear analysis, as shown in Figure 8. Mean and standard deviation values can be found in Table 2 and Table 3. After 90 h, CFUs of both dilutions significantly decreased for 5, 10, and 20 µg/mL gentamicin treatments, as shown in Figure 9. Kinetic differences in the resazurin assay were not dose-dependent, likely due to the complexity related to monitoring metabolic activity, stress, and growth at the same time, as shown in Figure 10. The stress-related increase in metabolic activity relative to comparisons between 0 minutes and 90 minutes was consistent across all of the concentrations of gentamicin except for the 0.1 µg/ml dose, as shown in Figure 11A. This variation in gentamicin effects in the resazurin assay could be due to the phase of metabolism of the pathogen. The cyclical phase of *M. avium* demonstrates the highly dynamic nature of metabolic energy released by the pathogen, as shown in Figure 11B. The non-treated *M. avium* is shown in blue, while the highest dose of gentamicin is shown in red. The stress and impact of the gentamicin over time is evident by the higher values of relative fluorescent units (RFUs) in the culture, which by 90 h are comparable to just the stress of the culture itself. Each of the concentrations of gentamicin ultimately had an impact on the metabolic stress of the bacterial culture, which in the evaluation of the beginning RFUs to the end RFUs was significant, as shown in Figure 11A versus Figure 11B.

**Figure 12:**
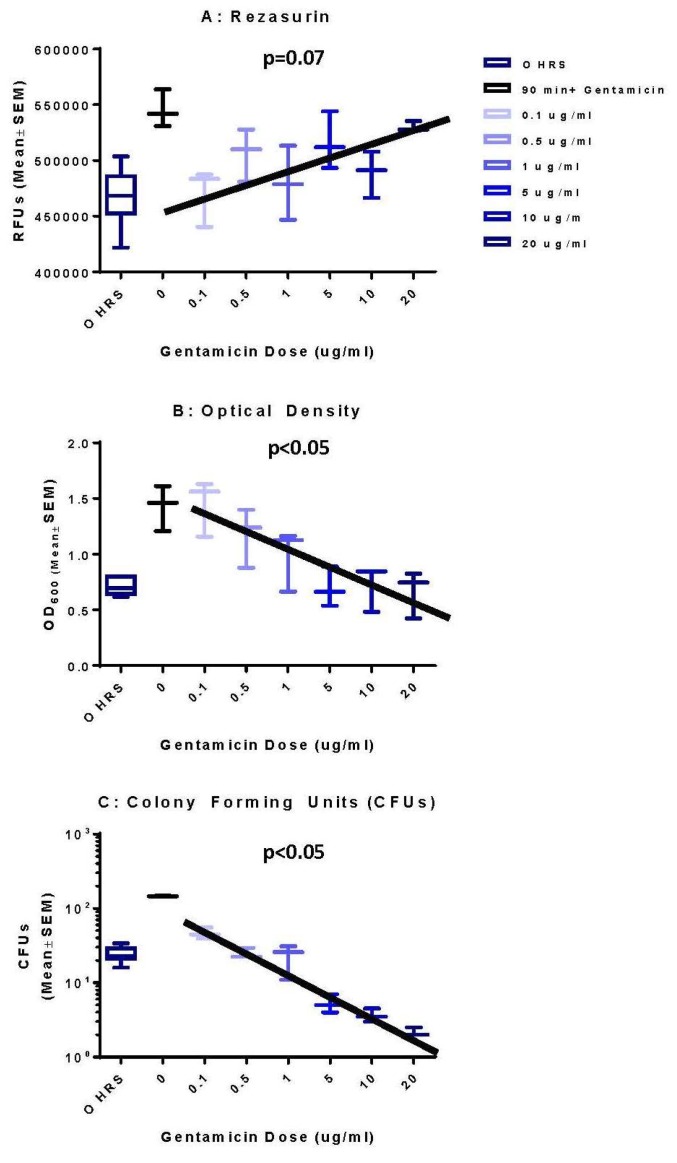
Linear regression comparison between the resazurin, OD, and CFUs of growing *M. avium* in the presence and absence of dose escalation of gentamicin. The comparison is between the 0 time point and 90 h and the impact of the different concentrations of gentamicin over the course of the 90 h. (**A**) Linear increase of resazurin as a component of enhanced stress, sub-statistical at *p* = 0.07. (**B**) OD as a component of fewer numbers of bacteria over time, *p* < 0.05. (**C**) CFUs demonstrating the surviving bacteria cultured at the end of each time point, *p* < 0.05.

In the final evaluation of the methodologies, a direct comparison was made between the metabolic stress levels of the bacteria, as shown in Figure 12A, the total number of bacteria as defined by OD, as shown in Figure 12B, and surviving bacteria as defined by CFUs, as shown in Figure 12C, focusing on *M. avium*. In this comparison, the metabolic stress was enhanced with the addition of the gentamicin in a dose-dependent fashion, whereas the total number and surviving bacteria slowly decrease as markers of less capacity to grow and less available to culture. These correlations are significant at *p* < 0.05 in each comparison, with a greater relation *p* < 0.01 for OD versus CFUs.

## 4. Discussion

New methods are desperately needed to evaluate therapeutics to treat *MAC* and the bacterial species it comprises. The developed methodology outlined here provides a quick and effective way to observe the growth patterns of NTM, specifically those involved in *MAC*. The studies outlined in this manuscript successfully developed new protocols for monitoring growth of *MAC* species *M. avium* and *M. intracellulare* in a timeframe useful for therapeutic development and testing. These studies demonstrate the successful combination of both solid and broth media to enhance the growth of *M. avium* and *M. intracellulare* while still enabling observation of colony growth and morphology. Growing the bacteria in both supplemented broth and solid media allowed for the performance of weekly studies, illustrating its effectiveness in speeding up the typical growth time of these strains. During a 90-hour study, optical density and cellular activity can be measured at 11 time points and CFUs can be measured at five time points. Optical density data was measured immediately at each time point while the cellular activity of samples collected at each time point were measured at the end of 90 h, using the resazurin assay. CFUs reflected the successful survival of the pathogens in the new growth protocols and show how the CFUs can be utilized for therapeutic testing and development. Although more detailed and specific technological approaches can be utilized with molecular biologic approaches, the need for a simplistic technology to monitor therapeutics over a variety of variables is essential for the initial clinical sensitivity testing. The pursuit of *MA*C characterization using the four methods outlined in this manuscript to observe bacterial growth and activity optical density (OD), colony-forming units (CFU), the resazurin assay, and fluorescent labelling, provide the foundation for protocols which can be easily used to monitor new therapeutics.

Optical density is used to measure bacterial growth in real time [20]. In these studies, measuring OD at each time point demonstrated that *M. avium*, *M. intracellulare*, and *MAC* can be monitored over a 90-hour experiment window. A comparison of OD at zero and 90 h indicated that there was significant growth of each bacterial strain independently and when combined at a p-value less than .01. The OD can indicate the likelihood of bacterial growth on agar plates for determination of *MAC* CFUs. However, OD cannot accurately predict bacterial count on its own and often underestimates growth once bacteria reach maximum cell density, as defined by “too numerous to count” (TNTC) [20,21].

To supplement data collected using OD, CFUs were calculated using a serial dilution and plate counting technique, determining bacterial growth after a one-week incubation period. This method is accurate even when accounting for dilution errors up to 10% [22]. By plating bacteria at time points throughout the experiments, CFUs were calculated and analyzed to create bacterial growth curves for *M. avium*, *M. intracellulare*, and *MAC*. Comparing different dilutions of the bacteria provided insight towards the optimization of CFU counts and growth curve kinetics. CFU counts at 10^-6^ dilutions revealed increased growth of *M. intracellulare* over the 90-hour experiment at a significant level of .01. Similar growth trends were observed at 10^−7^ dilutions for *M. avium* and *MAC*. Diluting the bacteria to 10^−7^ also demonstrated increased growth for *M. intracellulare*, but at a p-value of .05. These observations are consistent with the OD data, indicating overall growth over a 90-hour period, with *M. avium* exhibiting a strong influence over this trend. *M. avium* had higher CFU overall and *MAC* displayed increased counts when compared to *M. intracellulare* grown independently.

Bacterial activity was also measured using the resazurin assay, which allows for observation of small volumes of solution without the use a spectrometer [23]. The resazurin assay measures growth by detecting fluorescence as an indicator of cellular metabolic activity [21]. Resazurin data indicated that metabolic activity of *M. avium*, *M. intracellulare*, and *MAC* followed a cyclic pattern resulting from intra- and intercellular activity within the bacterial milieu. This pattern may be an indicator of the cell cycle, with higher fluorescence levels correlating to DNA replication and bacterial propagation and lower levels correlating to dormant phases of the cycle. The detected fluorescence results from the ability of metabolically active cells to reduce the resazurin dye to resorufin and dihydroresorufin, indicating their viability as a measure of cell proliferation [24]. After 90 h, activity significantly increased for the bacterial flasks containing *M. avium*, consistent with OD and CFU data that suggest the strain’s stability and dominance both grown independently and when combined (in *MAC)*. The overall activity of *M. intracellulare* appeared to decrease over the same duration of time.

A combination of using OD, CFU, and the resazurin assay allowed for comprehensive measurement of bacterial growth and metabolic activity and comparison between the three methods. Measuring OD and CFU provided similar data, a quantification of growth of each bacterial strain over the 90-hour period. The resazurin assay provided metabolic activity of each strain over the course of the experiment, suggesting growth fluctuations related to the cell cycle. Statistical analysis of these three tests indicates that significant bacterial growth occurred for *M. avium*, *M. intracellulare*, and *MAC*. Each methodology provided a unique way to characterize the growth patterns of these bacterial strains and together provide a more complete methodology with which to measure growth kinetics and monitor anti-*MAC* therapeutics in a short, replicable period. The application of the techniques to explore therapeutics was evaluated using the *M. avium* response to gentamicin confirmed assay sensitivity and the ability to evaluate growth kinetics in response to therapeutic treatment. *M. intracellulare* and *MAC* had similar growth profiles as defined by the extensive studies outlined here and have similar predictive levels of sensitivity and specificity. In the *M. avium* studies, stunted OD growth was observed as the gentamicin dose was increased, indicating less available bacteria after 90 h. Decreased CFUs confirmed decreased growth and viability of *M. avium* with higher doses of gentamicin. However, resazurin data indicated that cellular metabolic activity was not dose-dependent like OD and CFUs, but still changed according to different gentamicin treatments. Since gentamicin’s antibiotic activity stems from its ability to block protein translation through ribosome binding [25], higher doses of gentamicin would further obstruct protein translation and stunt growth, explaining the decreased OD and CFU values for the gentamicin dose-response study. NTMs are sensitive to gentamicin, and although it is not the most potent antibiotic in the treatment arsenal, it was used to define the sensitivity and specificity of a gentamicin response [26]. Other treatment options being currently explored include aminoglycosides, clofazimine, bedaquiline, and dapsone, however each of these have varying levels of effectiveness against *M. avium*, *M. intracellulare,* and *MAC* [26,27,28]. NTMs in chronic lung diseases do not infect these patients as singular entities; other pathogens often treated with gentamicin, such as *Pseudomonas aeruginosa* co-colonize [29,30]. Cellular metabolic activity, as detected by the resazurin assay, may not be dose dependent because, rather than testing for bacterial growth and viability, it detects metabolic activity of the remaining bacterial cells [28,31]. The cyclic nature of this activity could reveal opportune times to give therapeutics based on metabolic timing or the bacteria’s growth kinetics overall. The ability to quantify growth and metabolic activity of mycobacterial strains will be important in determining type of treatment, dosage, and timing of drug delivery as well as further characterization of *MAC* and other NTM diseases [26]. The ability to detect gentamicin sensitivity and specificity in the protocols validates the capacity to utilize these methods for therapeutic development. It is of importance to note that gentamicin inhibitory activity on *M. avium* is pH dependent, adding another layer of complexity to NTM ongoing studies. In this manuscript, all studies were carried out at the homeostatic pH of 7.5 as a baseline for characterization, however, alterations of these culture conditions could change antibiotic impact as well as baseline growth kinetics [32,33].

Given the rapid development of new strains of NTM and their varying clinical presentations, initial clinical sensitivity testing that is fairly rapid and straightforward to carry out is contraindicated [28,30]. The success of this protocol in observing the individual mycobacterial strain growth and as part of *MAC* suggests that it could be used to differentiate additional mycobacterial strains that comprise *MAC* and offer new directions for therapeutic studies aimed at attenuation of the disease [31,33].

## Figures and Tables

**Table 1:** Mean and standard deviation of optical density (OD_600_) for *M. avium* with gentamicin treatment over 90 h, *n* = 3.

Time (h)	0 µg/mL		0.1 µg/mL		0.5 µg/mL		1 µg/mL		5 µg/mL		10 µg/mL		20 µg/mL	
	Mean	SD	Mean	SD	Mean	SD	Mean	SD	Mean	SD	Mean	SD	Mean	SD
0	0.699	0.090	0.703	0.082	0.715	0.081	0.719	0.080	0.710	0.087	0.711	0.081	0.702	0.089
18	1.193	0.344	1.012	0.068	0.980	0.039	1.000	0.041	1.016	0.031	1.009	0.021	0.990	0.040
21	1.220	0.296	1.067	0.070	1.011	0.029	1.025	0.036	1.035	0.018	1.027	0.007	1.003	0.024
24	1.205	0.213	1.103	0.064	1.018	0.010	1.027	0.042	1.028	0.036	1.013	0.042	0.987	0.036
42	1.286	0.055	1.288	0.035	1.092	0.081	1.059	0.133	0.956	0.142	0.957	0.159	0.873	0.122
45	1.365	0.148	1.370	0.130	1.167	0.049	1.129	0.022	1.031	0.042	1.009	0.040	0.945	0.059
48	1.376	0.148	1.398	0.165	1.184	0.083	1.137	0.021	1.037	0.008	1.017	0.030	0.931	0.069
66	1.384	0.171	1.417	0.210	1.163	0.199	1.065	0.159	0.902	0.094	0.887	0.126	0.802	0.105
69	1.374	0.166	1.392	0.205	1.144	0.213	1.049	0.168	0.861	0.110	0.851	0.139	0.780	0.120
72	1.371	0.185	1.395	0.232	1.136	0.239	1.026	0.202	0.846	0.147	0.820	0.177	0.749	0.145
90	1.426	0.204	1.450	0.256	1.171	0.266	0.985	0.278	0.763	0.196	0.726	0.210	0.664	0.214

**Table 2:** Mean and standard deviation of bacterial growth (CFU) at 10^6^ dilutions for *M. avium* with gentamicin treatment over 90 h, *n* = 3.

Time (h)	0 µg/mL		0.1 µg/mL		0.5 µg/mL		1 µg/mL		5 µg/mL		10 µg/mL		20 µg/mL	
	Mean	SD	Mean	SD	Mean	SD	Mean	SD	Mean	SD	Mean	SD	Mean	SD
0	287.2	31.8	291.7	27.0	275.0	32.4	286.0	8.5	294.5	18.5	253.7	39.3	260.0	33.8
18	435.8	111.1	369.8	112.9	371.2	113.0	291.2	26.3	216.7	70.2	186.2	80.4	150.7	64.8
42	500.0	0.0	443.2	98.4	439.2	105.4	270.3	24.5	88.5	25.1	65.3	35.5	53.5	19.3
66	500.0	0.0	398.8	88.9	316.2	31.8	250.7	46.0	60.8	23.8	37.3	16.2	29.7	8.1
90	454.5	78.8	374.8	12.1	260.8	20.3	227.0	68.8	49.2	21.4	38.0	23.4	20.7	7.3

**Table 3:** Mean and standard deviation of bacterial growth (CFU) at 10^7^ dilutions for *M. avium* with gentamicin treatment over 90 h, *n* = 3.

Time (h)	0 µg/mL		0.1 µg/mL		0.5 µg/mL		1 µg/mL		5 µg/mL		10 µg/mL		20 µg/mL	
	Mean	SD	Mean	SD	Mean	SD	Mean	SD	Mean	SD	Mean	SD	Mean	SD
0	25.5	3.5	29.2	6.3	26.3	3.4	28.2	5.3	27.8	2.8	20.8	2.0	20.0	3.0
18	87.7	78.8	36.5	7.9	31.0	3.0	36.7	9.8	19.3	6.9	15.8	10.6	13.3	5.0
42	68.7	8.7	46.5	20.0	39.7	7.3	31.2	10.7	9.7	3.3	5.7	1.6	6.2	2.8
66	97.0	28.8	33.5	5.2	31.7	6.3	24.2	4.5	6.3	1.4	3.5	1.3	3.0	1.0
90	113.0	57.7	46.5	8.2	24.8	4.0	22.8	10.6	4.8	2.3	3.3	1.3	2.2	0.3
